# Relationship of Overweight and Obesity with Body Self-Image Dissatisfaction in Urban Mediterranean Adolescents

**DOI:** 10.3390/ijerph18157770

**Published:** 2021-07-22

**Authors:** Dolores Escrivá, Esther Moreno-Latorre, Jordi Caplliure-Llopis, Inmaculada Benet, Carlos Barrios

**Affiliations:** 1 Intensive Care Unit, La Fe Polytechnic and University Hospital, 46026 Valencia, Spain; dolores.escriva@ucv.es; 2 Nursing Department, School of Medicine and Health Sciences, Valencia Catholic University, 46001 Valencia, Spain; 3 School of Psychology and Education Sciences, Valencia Catholic University, 46110 Godella, Spain; esther.moreno@ucv.es; 4 Institute for Research on Musculoskeletal Disorders, School of Medicine and Health Sciences, Valencia Catholic University, 46001 Valencia, Spain; jorcallo@mail.ucv.es; 5 Primary Health Care Services, La Ribera University Hospital, 46600 Alzira, Spain; 6 Surgical Nursing Division, Valencia Clinic Hospital, 46010 Valencia, Spain; Inmaculada.benet@ucv.es

**Keywords:** body image, adolescence, anthropometry, weight status, overweight, obesity

## Abstract

The aim of this study was to analyze whether weight status has a relationship with the prevalence of body self-image dissatisfaction in Mediterranean urban teenagers. A series of 809 adolescents aged 11 to 17 years underwent anthropometric measurements according to ISAK protocols and completed the Body Shape Questionnaire (BSQ). The overall overweight prevalence according to International Obesity Task Force (IOTF) criteria was 11.5%, and 2.7% for obesity. Girls showed higher overweight prevalence than boys (18.4% vs. 12.9%; *p* < 0.05). At the late adolescence period (16–17 y), obesity was observed in the boys but not in the girls (8.7% vs. 0%; *p* < 0.01). There was a relative low prevalence of body image (BI) dissatisfaction among participants (boys 17.3%; girls 22.7%). In the late adolescence period, the girls were more often classified as being dissatisfied (31%). A weak correlation between the BSQ scores and all the anthropometric variables related to the adiposity profile was detected only in the boys. A logistic regression confirmed that female adolescents and the late pubertal period had a significant association with body dissatisfaction, regardless of their weight status. As BI are not related to weight status measured by body mass index (BMI) percentiles, other factors beyond anthropometry deserve further research to explain BI concerns specifically in girls.

## 1. Introduction

For more than three decades, overweight and obesity in childhood and adolescence have been considered one of the most relevant public health challenges in developed countries [1,2]. The most recent WHO estimates show that overweight and obesity prevalence among children and adolescents aged 5–19 years has risen considerably [3]. In about 40 years (1975–2016), overweight prevalence passed from just 4% to over 18%, equally affecting both boys and girls.

According to the latest report delivered in 2017 by the Non-Communicable Disease Risk Factor Collaboration [4], the largest database on obesity in childhood and adolescence, the prevalence of obesity between 1975 and 2016 rose from 0.7% to 5.6% in girls and from 0.9% to 7.8% in boys. The highest increase was detected in English-speaking and Mediterranean countries. In fact, high overweight and obesity prevalence was recently found in children and adolescents residing in Southern Europe (the Southeast Spain and Southern Italy regions): 34.8% for boys and 29.9% for girls, according to the International Obesity Task Force (IOTF) cutoff measures [5].

In Spain, overweight and obesity prevalence has shown a substantial and continuous increase, specifically during childhood and adolescence [6]. Overweight and obesity prevalence data from Mediterranean urban areas is still needed.

Overweight and obesity during childhood and early adolescence have multiple well-documented negative consequences for immediate and future health [7]. The impact of overweight and obesity on morbidity covers a variety of conditions, from metabolic and cardiovascular disease, type 2 diabetes, some cancers, and musculoskeletal disability to premature death [7,8,9]. However, the negative psychological implications of childhood obesity still require more attention. Children with overweight or obesity or and adolescents could also develop a series of psychological comorbidities, including emotional and behavioral disorders, a tendency toward depression, anxiety, and low self-esteem [10,11].

Adolescence is a period of intense biopsychosocial maturation [12]. The most relevant changes during this crucial development period are related to body morphological transformations in both sexes [13]. These critical changes in physical appearance can considerably influence adolescents’ self-appraisal of body image (BI) [14]. This self-concept of BI is a multidimensional construct involving the accuracy of the person’s perception of the shape and size of their body along with the feelings that this representation can cause [15]. Specifically, in childhood and adolescence, a relevant factor influencing the development of self-concept and self-esteem is BI, which can be negatively affected in obesity [15,16,17,18].

Children and adolescents experiencing BI disturbances often claim dissatisfaction with their body shape and/or weight [19,20]. In a relatively recent review analyzing differences in BI between individuals with normal weight and those with obesity, increased body dissatisfaction was observed among the latter, especially among women [21]. Although most of the research in this topic has been focused on adults who are overweight or with obesity, children and adolescents are also able to claim weight concerns and body dissatisfaction [22,23].

The consequences of overweight and obesity on BI disturbances are less explored than those of other health-related factors. To evaluate negative feelings regarding BI in adolescents, different instruments have been culturally adapted. Self-assessment questionnaires are best suited to evaluate the components of BI in both epidemiological and clinical studies [24,25]. One of these instruments is the Body Shape Questionnaire (BSQ) [26], which is aimed at exploring BI self-perception. The BSQ is one of the most used tools in research concerning the psychological impact of body characteristics in the adolescent population at the national and international levels. BSQ validations have been conducted in several populations, yielding data that confirms the excellent level of internal consistency in its original form. Raich et al. [27] adapted and validated the instrument to the Spanish population, showing high internal consistency.

Our hypothesis is that in urban Mediterranean adolescents body dissatisfaction assessed by the BSQ instrument might be more related to sex differences in adolescents’ self-appraisal of body image than a particular weight status, such as overweight and obesity. To our knowledge, differences in body dissatisfaction between Mediterranean children and adolescents who have overweight and obesity and those with normal weight from the same region have never been explored and quantified to date. With this background data, the purpose of this study was to analyze whether the weight status of a sample of urban Mediterranean adolescents measured by body mass index (BMI) percentiles could have a relationship to their BI dissatisfaction. Changes in BI dissatisfaction in different periods of the adolescence were also studied and compared between sexes.

## 2. Patients and Methods

The Health Sciences Research Committee of the School of Doctorate of the Valencia Catholic University revised and approved the study. Its execution was accomplished in accordance with the ethical principles for medical research involving human subjects (Declaration of Helsinki of the World Medical Association).

### 2.1. Participants

The students were recruited from three secondary schools located in Valencia, a Spanish Mediterranean big city with more than 1 million inhabitants. The schools were representative of the most common population of the city in terms of middle-level socioeconomic conditions. Private schools with high scholarship fees were not considered for this investigation. In order to select the schools for this study, the researchers arranged a meeting with the board of directors from more than eight similar schools in which the aim and the details of the project were presented. The three selected schools were the first to approve the proposed research study.

Prior to voluntarily joining the study, all the possible participants and parents were fully informed about the objectives of the investigation. Initially, the research project was presented to a total sample of 1149 secondary students. The 70.4% of students gave their assent to participate in the study, and their parents gave informed written consent. Among the final 809 participants, 413 (51.1%) male and 396 (48.9%) female were included in the study. The mean and standard deviation (SD) of age was 13.8 ± 2.0 years and 13.7 ± 1.9 years for the males and the females, respectively.

### 2.2. Anthropometric Measures and Weight Status

Common anthropometric parameters such as stature and body mass were recorded following standardized methods [28]. Age and sex were self-reported, and the classification of BMI (body mass [kg]/height [m]^2^) was used as the primary assessment of weight status. Weight status was categorized as underweight, overweight, and obesity according to the International Obesity Taskforce (IOTF) criteria, which provide sex- and age-specific BMI cutoffs based on BMI percentiles [29,30]. The study employed the WHO categorization of adolescence periods [31]: the early adolescence period (EAP), 10–13 years; the middle adolescence period (MAP), 14–15 years; and the late adolescence period (LAP), 16 years or more.

### 2.3. Body Self-Image Evaluation

The instrument selected for BI assessment was the BSQ [7], with a transcultural adaption for Spanish adolescents [27]. The BSQ is a self-administered questionnaire consisting of 34 questions in a Likert-like self-reporting scale, with six response options (i.e., 1 = never, 2 = rarely, 3 = sometimes, 4 = often, 5 = very often, 6 = always). These questions cover four domains: body dissatisfaction, 12 questions; fear of gaining weight, seven questions; feelings of worthlessness because of appearance, 10 questions; and desire to lose weight, six questions. Four levels of dissatisfaction with physical appearance were considered and scored as follows: free of body dissatisfaction, below 80 points; mild dissatisfaction, 80–110 points; moderate dissatisfaction, 110–140 points; and severe dissatisfaction, greater than or equal to 140 points [32].

The decision to use this questionnaire was based on several criteria: adaptation to the Spanish population, specificity to the assessment of body dissatisfaction among adolescents, brevity, and ease of application. The reliability of this questionnaire has been secured, with high internal consistency obtained in the study of adaptation (Cronbach’s alpha between 0.95 and 0.97) [27].

### 2.4. Statistical Analysis

Anthropometric data and scores from the questionnaires were evaluated using statistical analysis software (SPSS Inc. v.20, Chicago, IL, USA). A descriptive analysis was performed using frequency, mean, and standard deviation (SD). For contrast analysis, parametric tests were selected. The variation in scores of BI dissatisfactions among the different periods of adolescence and among the groups of weight status was evaluated using the analysis of variance (ANOVA) test. We applied the *t*-test to compare the mean scores of the instruments between the sexes. The chi-square test, in the case of ordinal factors, and Fisher’s exact test for the nominal factor were applied to examine the associations of the adolescence periods (early, middle, and late), sex, and weight status as well as the frequency of subjects classified as satisfied and dissatisfied by the BSQ. The effect sizes were calculated using Cohen’s d method with the following thresholds: small (d = 0.20–0.49), medium (d = 0.50–0.79), and large (d > 0.80) [33]. Pearson correlation coefficients were used to determine the relationships among the continuous variables. The strength of correlation was defined as trivial (r = 0–0.1), small (r = 0.1–0.3), moderate (r = 0.3–0.5), large (r = 0.5–0.7), very large (r = 0.7–0.9), or nearly complete (r = 0.9–1.0). The level of probability (*p*-value) was considered statistically significant for values <0.05.

A model of binary logistic regression was adjusted to determine which of the predictive variables had significant associations with the risk body dissatisfaction. To meet the response variable’s requirement for dichotomy, the BSQ was reorganized into two categories: satisfied (classified as free of body dissatisfaction) and dissatisfied (with some level of dissatisfaction: mild, moderate, or severe). The same was done for the weight status variable, which had its four categories grouped into two: underweight/eutrophic and overweight/obesity. Adolescence periods were also dichotomized into two categories, early-middle and late adolescence.

## 3. Results

### 3.1. Weight Status

**Figure 1:**
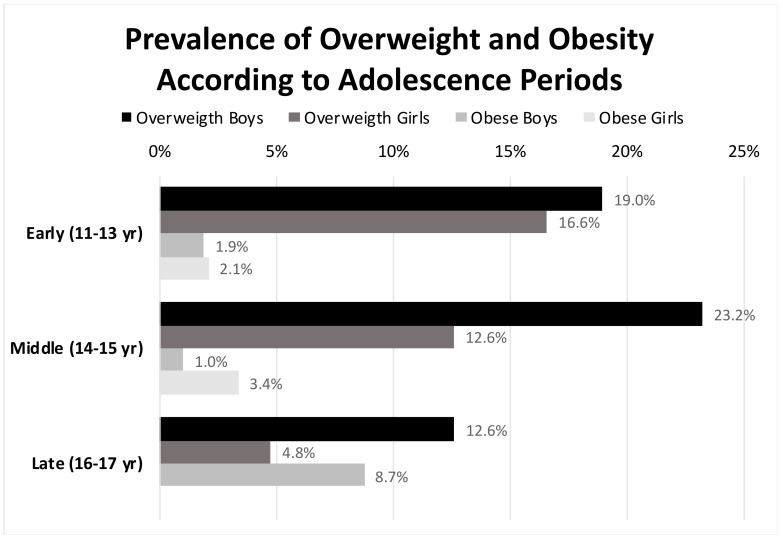
Changes in the prevalence of overweight and obesity in girls and boys along the three periods of the adolescence.

As for the weight status, 5.8% of the participants were underweight, 15.7% overweight, and 2.7% had obesity. Considering the whole sample, there were differences between the girls and the boys in terms of overweight prevalence, higher for the boys (12.9% vs. 18.4%; chi-square test = 4.792; *p* = 0.028). The underweight prevalence was slightly higher in the girls than in the boys (6.6% vs. 5.1%) without statistical significance. The prevalence of obesity was quite similar in both sexes (2.0% in girls and 3.4% in boys). Figure 1 represents the changes in the proportion of girls with overweight and obesity and boys within the three adolescence periods. At the MAP, the overweight prevalence was higher in the boys than in the girls (23.2% vs. 12.6%; chi-square test = 4.421; *p* = 0.039). At the LAP, obesity was found among the boys but not in the girls (8.7% vs. 0%; chi-square test = 7.711; *p* = 0.004). Overweight and obesity prevalence in the girls showed a progressive decrease along the adolescence periods. The boys’ obesity prevalence increased significantly from the MAP to the LAP (from 1.0% to 8.7%; chi-square test = 6.407; *p* = 0.011).

### 3.2. Body Shape Questionnaire (BSQ) Scores

The mean value of the BSQ scores in the whole sample was 58.9 ± 26.9 (95% confidence interval (CI): 57.1–60.8). The girls showed statistically significantly higher mean values than the boys (i.e., 61.7 ± 26.6 vs. 56.3 ± 27.1; t = 2.849, *p* < 0.01; Cohen’s d = 0.20). The results concerning BSQ scores according to adolescence periods and sex are indicated in Table 1.

According to BSQ score stratification, 339 boys (82.7%) were satisfied with their BI, while 71 (17.3%) were dissatisfied, 54 (13.2%) with mild dissatisfaction, 10 (2.4%) with moderate dissatisfaction, and seven (1.7%) with severe body dissatisfaction. Among the girls, 304 (77.4%) were satisfied with their BI, while 89 (22.7%) were dissatisfied: 69 (17.6%) with mild dissatisfaction, 14 (3.6%) with moderate dissatisfaction, and six (1.5%) with severe body dissatisfaction. Non-significant differences were found in the distribution of BI dissatisfaction and sex.

Table 1 includes also the BSQ mean scores and the frequency of BI dissatisfaction among the three adolescence periods in both sexes. Differences were observed between the sexes in the mean scores of the BSQ in the EAP, with greater dissatisfaction in female than in male adolescents (t = −3.193; *p* < 0.01; Cohen’s d = 0.32). According to the BSQ’s classification, the girls in the LAP were more often classified as being dissatisfied (31%) than those in the EAP group (19.1%; chi-square test, *p* < 0.05). Regarding the boys, the LAP group also disclosed a higher percentage of BI dissatisfaction (22.3%), but the differences were not as significant as those of the other groups (EAP, 14.4%; MAP, 18.4%).

**Figure 2:**
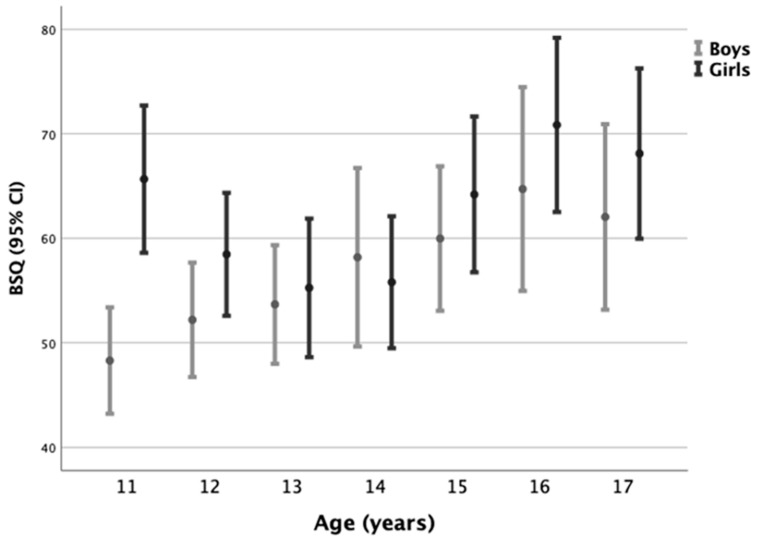
Body Shape Questionnaire (BSQ) mean scores at the different ages according to sex (Dots = mean values; CI = confidence interval).

A progressive increase in BSQ mean scores with age was observed in the boys until the age of 16 years. The girls showed an initial decrease in BSQ scores from ages 11 to 12 years (Figure 2). From 12 to 17 years, the BSQ scores of the girls exhibited the same behavior as those of the boys. Differences between sex were only found at 11 years of age, when the girls showed higher mean values (65.6 ± 25.3 vs. 48.3 ± 19.9); t = 4.079; *p* < 0.001; Cohen’s d = 0.78).

### 3.3. Correlations between BSQ and Anthropometry

While analyzing the whole sample, Pearson’s correlation coefficients between BSQ scores and the anthropometric variables were statistically significant except for the biceps skinfold (Table 2). When both sexes were analyzed separately, these correlations disappeared completely in girls, but they remained in boys. In girls, there was only a positive slight correlation of BSQ scores with age.

### 3.4. BSQ and Weight Status

**Figure 3:**
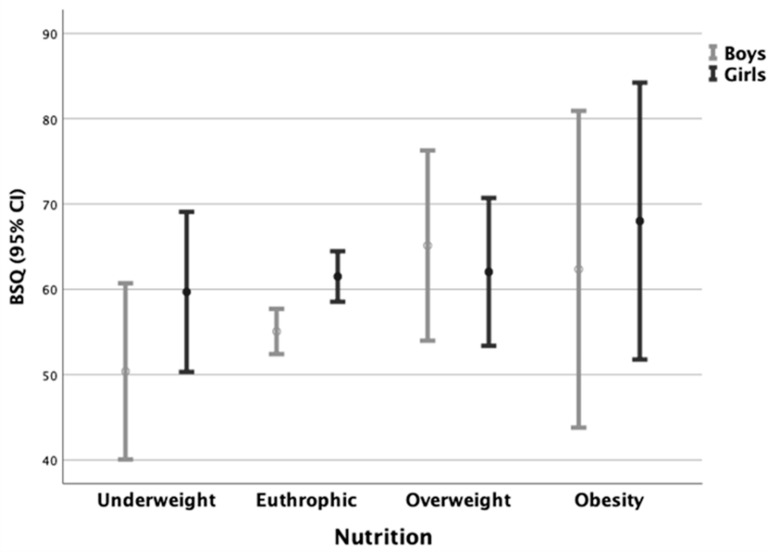
BSQ mean scores according to BMI weight status (Dots = mean values; CI = confidence interval).

When the BSQ’s mean scores of body dissatisfaction were analyzed according to the weight status of the adolescents, a greater trend toward more BI dissatisfaction was observed in overweight adolescents and those with obesity than in those with low BMI, without statistical significance (F_[df3]_ = 1.949, *p* = 0.120) (Figure 3). Comparing sexes, the BSQ mean values were more stable in the girls, passing from 59.7 ± 23.2 in underweight to 68.0 ± 31.5 in girls with obesity (Figure 3). Only the eutrophic group had statistically significant differences, with higher BSQ mean values in the girls (i.e., 61.5 ± 26.3 vs. 55.1 ± 22.7; t = −3.211; *p* < 0.001; Cohen’s d = 0.26). Interestingly, adolescents within the normal range of weight (19.5%) also expressed body dissatisfaction (chi-square test < 0.05). The lowest frequency of body self-image dissatisfaction was found in underweight boys and girls (14.3% and 15.4%, respectively).

Using multifactorial analysis including sex, weight status, and adolescence period, only this last variable was related to body self-image dissatisfaction (Wilks’ Lambda: 0.983; *p* = 0.004). The binary logistic regression model from the odds ratio (OR) risk estimator confirmed that girls (OR:1.19, *p* = 0.043) and the late adolescence period (OR:1.18, *p* = 0.012) had a significant association with body dissatisfaction.

## 4. Discussion

This cross-sectional study found that the estimated overweight and obesity prevalence in Mediterranean urban adolescents aged 11 to 17 years was 15.7% and 2.7%, respectively (IOTF guidelines). The prevalence was higher in the male participants (18.4% underweight; 3.4% with obesity) than in the female participants (12.9% underweight; 2.0% with obesity). Our data confirm that excess weight was more prevalent in the boys than in the girls during adolescence. These differences increased as the adolescence periods progressed, particularly for obesity since the boys showed a trend toward increased prevalence (opposite for the girls).

In Spain and other countries, information about weight excess during this specific age range (11–17 years) is still scarce. In addition, comparisons among prevalence in different studies and trends are difficult to track because some of these surveys have used distinct criteria to estimate prevalence and, thus, they have obtained different results. In 2005, overweight (and obesity) prevalence in a representative sample of Spanish adolescents was 25.7% in boys and 19.1% in girls [34]. In 2012, the obesity prevalence figures in the MAP and LAP (14–17 year) reached 6.7% using the IOTF criteria [6]. The prevalence of excess weight (overweight and obesity) of this study was lower than those recently reported for other Spanish non-Mediterranean urban populations aged 9 to 24 years within the ENPE study, also following IOTF guidelines [35]. Exploring a smaller population (193 boys and girls in the age range of 9–18 years), this survey observed significantly higher overweight (35.6% in boys; 22.4% in girls) and obesity (8.5% in boys; 5.3% in girls) prevalence than this study. Also, a recent cross-sectional study of the weight status of Spanish teenagers at the MAP revealed slightly lower overweight and obesity prevalence rates (4.3% and 3.0%, respectively, in girls; 4.7% for both overweight and obesity in boys) [36]. This last study, based on BMI data, used different percentiles for weight status classification than those proposed by the IOTF; however, the figures are closer to our results.

Using the BSQ cutoff of 80 points, the prevalence of body dissatisfaction in the current sample of Mediterranean teenagers was relatively low (17.3% and 22.7% for males and females, respectively) compared with those of other similar studies in other countries [37,38]. Adolescents with moderate (111–140 points) or extreme (>140 points) concern also showed lower prevalence that those of previous studies: 4.1% for males and 5.1% for females. Contrary to the previous data [38], when the current series was analyzed by age or adolescence period, a significant increase was observed in the mean BSQ scores with increasing age. Although this increase in BSQ scores existed in the boys, it was greater and more statistically significant in the girls. The relative deterioration of body self-image with age cannot be explained only by the increase in BMI that occurs between 10 and 17 years of age. At least, this does not apply for our sample of adolescent girls since those with moderate and extreme body dissatisfaction had similar BMIs to those with body satisfaction. Although the prevalence is higher in girls, these overall figures did not show any statistically significant difference between sex.

In our series, 31.0% of the girls in late adolescence (16–17 years of age) had concerns about BI compared with 18.1% of those in early adolescence (11–13 years). These data are in accordance with previous studies reporting evidence that older adolescents, especially girls, have a much higher prevalence of body dissatisfaction than do younger adolescents [39,40,41]. Lower body dissatisfaction in early adolescence could be related to the lack of BI identity because of morphological and biopsychosocial changes occurring at early adolescence [18]. Adolescent girls seem to increase their personal expectations after menarche, being more dissatisfied with the changes related to body fat accumulation and turning their attention to weight reduction [14,42,43]. Furthermore, the internalization of a thin body induced by the current culture may be one of the main factors influencing girls to dislike their physical appearances during late adolescence [44]. In boys, this effect may be less intense because they do not experience as much pressure to achieve an ideal body shape [45]. While the standard for the ideal female body is to be thin, the model for boys released by the current social atmosphere is a muscular and fat-free body [46,47].

Given the high prevalence described in the literature, some authors consider the occurrence of body dissatisfaction among girls as “normative” [38,39]. However, in our sample of Mediterranean adolescent girls, concern about body dissatisfaction was only identified in less than one third of the cases. On the other hand, the maturing process of adolescent boys differs from that of girls. The main characteristic of male adolescence is the progressive decrease of body fat and the corresponding increase of fat-free mass [48]. The increase in lean mass that occurs particularly during adolescence in boys can be considered a protective factor against the development of feelings and/or thoughts of aversion against the body itself [40]. Previous studies postulated that body dissatisfaction seems to diminish throughout adolescence among adolescent boys [39,40,41,46,48]. However, with regard to the different adolescence periods analyzed, the BSQ scores obtained from the boys in this study do not corroborate the previous findings. In our series, the boys’ BSQ scores also increased as age increased. In fact, 22.3% of the males between 16 and 17 years old had concerns about BI compared with only 14.4% in the age group of 11–13 years. Therefore, contrary to previous reports, body dissatisfaction in boys does not tend to decrease as puberty advances. More interestingly, in our series, the mean age of the boys with moderate or extreme body dissatisfaction was higher than that of the boys without dissatisfaction.

One study reported that girls are almost twice as likely to have body dissatisfaction in relationships with boys (BSQ-OR 2.893, *p* < 0.001) [23]. These authors observed that female teenagers tend to feel disproportionately fat, even when eutrophic or underweight, disfiguring the perception of body self-image. In agreement with this observation, our data also show that adolescents with overweight were more dissatisfied with their BI [47]. Remarkably, in our sample of healthy adolescents, worries about BI were related to higher BMI in boys but not in girls. Boys with extreme body dissatisfaction had a mean BMI of 25.5 ± 6.7 compared with 19.9 ± 3.3 in unconcerned boys. Perhaps this phenomenon could be related to the internalization of the muscular body commonly desired by boys [37,44,49]. Some authors claim that the ideal body can vary among different cultures [44,50]. In some cultural settings, increased muscle mass might not be good for BI in adolescent boys.

The results of this study indicate that body dissatisfaction is a reality in the lives of urban Mediterranean adolescents and that sex and morphological changes that occur during adolescence are factors related to such negative feelings of BI. The identification of factors that may have a relationship with BI dissatisfaction among adolescents seems, therefore, to be an important research issue, independent of the type and development of the population. These epidemiological studies, especially within school health developmental policy, are essential to provide information about teens’ dynamics, helping educators and health professionals design intervention strategies when needed.

## 5. Conclusions

In summary, this cross-sectional survey revealed that Mediterranean urban teenagers exhibit less overweight and obesity prevalence than expected according to the previously reported trend. The overall overweight and obesity prevalence was 11.5% and 2.7%, respectively. The girls showed a higher overweight prevalence than the boys (12.9% vs. 18.4%; *p* < 0.05). However, the prevalence of obesity was lower in the girls than in the boys (2.0% vs. 3.4%). Furthermore, regarding BI concerns, the prevalence of body dissatisfaction was also low: 17.4% for the boys and 22.7% for the girls. A significant increase was observed in the BSQ mean scores with increasing age, particularly in the girls. The binary logistic regression analysis confirmed that female adolescents and the late pubertal period had a significant association with body dissatisfaction. However, BI dissatisfaction was not related to weight status. In addition, the BSQ scores correlated positively with some anthropometric parameters related to adiposity, but these relationships only applied for the boys, not for the girls. Some other psychosocial factors beyond anthropometry merit further research to explain the lack of correlation between BI concerns and weight status specifically in girls.

### 5.1. What Do We Already Know about This Topic?

Children and adolescents with overweight or obesity are prone to develop psychological comorbidities, including emotional and behavioral disorders, anxiety, and low self-esteem.

### 5.2. How Does Your Research Contribute to the Field?

This study provides data concerning the weight status of schoolchildren and adolescents aged 11–17 years of a large urban Mediterranean city. Although the prevalence of overweight and obesity can be considered high in this population, the weight status has no relationship with body dissatisfaction. However, female adolescents and the late pubertal period had a significant association with body dissatisfaction. Apart from sex and morphological changes that occur during adolescence, other factors of the psychology environment could be related to negative feelings of body self-image.

### 5.3. What Are the Implications of Your Research in Terms of Theory, Practice, or Policy?

Some intervention strategies are needed to explore and minimize the impact of different psychosocial factors beyond anthropometry on body self-image.

## Figures and Tables

**Table 1:** Descriptive analysis of the anthropometric variables for periods of adolescence.

	Boys				Girls			
	EAP *n* = 211 Mean ± SD	MAP *n* = 99 Mean ± SD	LAP *n* = 103 Mean ± SD	*p* *	EAP *n* = 193 Mean ± SD	MAP *n* = 119 Mean ± SD	LAP *n* = 84 Mean ± SD	*p* *
BSQ Scores	51.5 ± 23.0	59.3 ± 26.4	63.3 ± 33.0	0.001	59.3 ± 26.2	60.1 ± 26.6	69.7 ± 26.4	0.001
BSQ Classification, *n* (%)								
Satisfied	179 (85.6)	80 (81.6)	80 (77.7)		158 (81.9) ^§^	88 (75.9)	58 (69.0)	
Mild Dissatisfaction	25 (12.0)	13 (13.3)	16 (15.5)		24 (12.4)	24 (20.7)	21 (25.0) ^§§^	
Moderate Dissatisfaction	4 (1.9)	4 (4.1)	2 (1.9)		7 (3.6)	3 (2.6)	4 (4.8)	
Severe Dissatisfaction	1 (0.5) **	1 (1.0)	5 (4.9)		4 (2.1)	1 (0.9)	1 (1.2)	

EAP: early adolescence period; MAP: middle adolescence period; LAP: late adolescence period; BMI: body mass index; FMI: Fat mass index; SD: standard deviation. * ANOVA test; ** Chi-square test; *p* < 0.05 as compared to AP of the other sex. ^§^ Chi-square test; *p* < 0.01 as compared to LAP of the same sex. ^§§^ Chi-square test; *p* < 0.05 as compared to EAP of the same sex.

**Table 2:** Correlations between BSQ scores and anthropometric variables (BMI = body mass index, FMI = fat mass index).

	Boys (*n* = 410)		Girls (*n* = 393)		Total Sample (*n* = 813)	
	Pearson’s Correlation	Sig. (Bilateral)	Pearson’s Correlation	Sig. (Bilateral)	Pearson’s Correlation	Sig. (Bilateral)
Age	0.192	<0.001	0.102	0.043	0.148	<0.001
BMI	0.213	<0.001	0.065	0.199	0.139	<0.001
Body mass	0.260	<0.001	0.077	0.129	0.170	<0.001
Calf skinfold	0.115	0.020	0.034	0.501	0.093	0.009
Thigh skinfold	0.119	0.016	0.032	0.530	0.099	0.005
Abdominal skinfold	0.147	0.003	0.019	0.710	0.098	0.005
Suprailiac skinfold	0.163	0.001	−0.007	0.894	0.095	0.007
Triceps skinfold	0.130	0.008	−0.015	0.773	0.079	0.025
Biceps skinfold	0.125	0.011	−0.011	0.824	0.065	0.066
Σ 6 skinfolds	0.154	0.002	0.003	0.954	0.100	0.005
Fat mass	0.246	<0.001	0.024	0.634	0.151	<0.001
Fat percentage	0.161	0.001	−0.016	0.752	0.093	0.009
FMI	0.198	<0.001	0.013	0.805	0.119	0.001

## Data Availability

Raw data are available upon request to corresponding author.
